# 

**Published:** 1911-06-15

**Authors:** 


### ANNOUNCEMENTS.

### NATIONAL DENTAL ASSOCIATION.

The Fifteenth Annual Session of the National Dental Association will be held in Cleveland, Ohio, July 25th to 28th and the indications are favorable for this being the best meeting the National has ever held.

Cleveland is a beautiful city, of 600,000 population, with exceptionally good rail and water transportation facilities; ample and convenient hotel accommodations; well arranged halls for meetings, clinics and exhibits, extensive and beautiful boulevards and parks, and a local Dental Society, with 140 members, actively engaged in making preparation for every convenience and comfort, of those who may attend, which goes far in making a successful meeting.

The complete program will be published in the July Dental Journals, but will state that the clinic Committee has given assurance that they will have some 300 clinics, and the following have been secured as essayists: Drs. S. G. Perry, New York City, R. H. Hofheinz, Rochester; C. N. Johnson, C. S. Case and Frederick B. Noys, Chicago; Charles S. Butler and F. W. Lowe, Buffalo; Joseph Head and J. G. Lane, Philadelphia, W. R. Clack, Mason City, Iowa; E. M. Kettig, Louisville; Alfred G. Fones, Bridgeport, Conn; George H. Wright, Boston; R. W. Bunting, Ann Arbor, Mich.; W. O. Hulick and W. H. O. McGehee, Cincinnati, and Varney E. Barnes, Cleveland.

Wednesday evening, July 26th, is to be devoted to an oral Hygiene meeting and Dr. Eugene H. Porter, New York State Commissioner of Health, has been invited to address this session.

The Revision Committee has called a meeting for 9 A. M. July 24th, at the Hollenden Hotel, and have invited every State Society and National Dental Organization, to send a representative to meet with them.

Railroad rates of a fare and a half, certificate plan, will be granted.

The local committee seem confident that we should have at, least 3000 dentists in attendance and are making arrangements accordingly. When we consider the favorable conditions, as outlined, we trust their expectation may be more than realized.

The interest of yourself and clientele are BEST served when YOU accept every such opportunity for professional advancement; therefore, YOU cannot afford to miss this meeting. General information concerning the local arrangements can be obtained by addressing Dr. Geo. H. Wilson, Chairman, 701 Schofield Bldg., Cleveland. Hotel reservation can be made through Dr. H. C. Kenyon, 730 Rose Bldg., Cleveland.

Membership in your state dental society, or allied societies, make you eligible to the National; membership fee $5.00. Application blanks may be secured from the secretaries of the state societies, or from Dr. Charles W. Rodgers Cor. Sec'y, 165 Harvard St., Dor., Boston, Mass.

Fraternally,

Homer C. Brown, *Rec. Sec'y.*

Edward S. Gaylord, *President.*

### CHICAGO DENTAL SOCIETY CELEBRATION.

This Society is already making large preparations to hold a two days meeting and clinic January 22 and 23, 1912. The Society has a membership of nearly 1200, probably the largest dental society in existence. If we are to judge from past experiences and the accustomed thoroughness of their methods and the early start they are making this will be a great meeting of dentists. The plan is to have both papers and clinics, and an effort will be made to present all the newest and best features obtainable. Make your plans thus early and reap all the benefits of such a grand meeting.

THE ILLINOIS STATE DENTAL SOCIETY held its forty-seventh Annual Meeting at Peoria, May 9-12, 1911.

The following officers were elected for the ensuing year: President, C. C. Corbett, Edwardsville; Vice-president, J. P. Luthringer, Peoria; Secretary, J. F. F. Waltz, Decatur; Treasurer, T. P. Donelan, Springfield; Librarian, A. Don Stocker, Alton.

The next Annual Meeting will be held at Springfield, May 14-17, 1912.

J. F. F. WALTZ, *Secretary.*

---

WISCONSIN STATE DENTAL SOCIETY. The next Annual meeting will be held July 11-13, 1911, at Eau Claire. A good programme is promised and a cordial invitation is extended to all ethical practioners.

---

### COME TO CLEVELAND FOR THE NATIONAL DENTAL ASSOCIATION.

JULY 25-26-27-28, 1911.

PROGRAM: A full literary program—Approximately 300 clinics—unusually large and varied dealers' exhibit—an ideal convention city, and your presence required to further the great interests confronting the profession of to-day; viz: State and National reorganization, Oral Hygiene, National Journal, and Good—fellowship.

RAILROAD RATES: Since the advent of the 2 cent fare, concessions have not been made for our National Meetings, but this year a concession of one and one-half fare (certificate plan) by the Central Passenger Association, with modifications by the other passenger associations, has been granted. Those residing in territory outside of the Central Passenger Association, should consult their ticket agent, and if satisfactory information cannot be obtained, write to Dr. G. D. Lovett, 761 Rose Bldg., Cleveland, Ohio, and he will instruct you.

As 1000 certificates are required and the number returned will much influence future concessions, it is desired that every one attending the meeting shall obtain a convention certificate with his railroad ticket, and present the same to the Information Bureau upon arrival. You are requested to do this whether you wish to return by the same route or not.

HOTELS: Write for a list of the principle hotels with their rates. YOU SHOULD WRITE THE HOTEL OF YOUR CHOICE AND MAKE RESERVATION EARLY. A large attendance is assured.

MEMBERSHIP: Any member of a State Association is eligible for membership in the National Association on certificate signed by the officers of his association. You are invited to become a member or to come as a guest. Make this a part of your vacation trip, a royal time is assured you. FOR INFORMATION ADDRESS:—

LOCAL COMMITTEE OF ARRANGEMENTS.
National Dental Association.
718 Schofield Bldg.,
Cleveland, Ohio.

### CALL OF ORAL HYGIENE CONFERENCE.

The Oral Hygiene Committee of the National Dental Association hereby issues a call for a conference on Oral Hygiene to be held in the Engineers' Building, Cleveland, O., Friday, July 28th, 1911, at 8 P. M.

This conference is called for the purpose of bringing together the Oral Hygiene workers of the Country with the object of discussing suitable ways and means of handling the Oral Hygiene problem of this Country in the most effective and economic manner.

The object of issuing a call for a meeting at this time and place is that the National Dental Association meets in Cleveland, O., July 25th to 28th, and the conference will be held immediately following the National Dental Association Meeting.

The Committee earnestly desires that every member of the Profession interested in the all important question of Oral Hygiene make it a point to be present at this meeting.

THE ORAL HYGIENE COMMITTEE OF
THE NATIONAL DENTAL ASSOCIATION
W. G. EBERSOLE, *Chairman.*
E. P. DAMERON,
RICHARD GRADY,
J. P. CORLEY,
H. C. THOMPSON,
B. HOLLY SMITH,
W. A. WHITE.

---

CORRECTION. In the May issue we printed an article by Dr. H. J. Goslee, which should have been credited to the *Western Dental Journal.* It was an error of the "*Devil*" not the Editor.

---

### A PERMANENT DEFENSIVE ORGANIZATION IS NECESSARY TO ASCERTAIN AND SAFEGUARD THE COMMON RIGHTS OF THE DENTAL PROFESSION.

The Dental Protective Association was formed and collected funds for the purpose of testing the validity of dental patents but has recently so changed its bylaws and strayed from its original purpose as to become the ally and collecting agency of a patantee whose test suit against the profession has been prolonged over the last three years: the patentee in the meantime securing two new patents to strengthen the claims of the original.

It was the patentee's own choice to apply the rigor of the law rather than an amicable and businesslike proposition to the profession. A defense committee was therefore organized and its work so successfully performed that there is every reason to believe the original Taggart patent will soon be decided in favor of the profession. But Dr. Taggart's attorney has recently announced the probable institution of suits against many dentists under the claims of the new patents whose validity each individual sued would have to bear the expense of testing in the courts unless we create a dependable permanent defensive organization.

The object of such organization should be safeguarded by proper limitations on the policy and powers of its managing officers. For instance, there should be no such concentration of authority as Dr. Crouse exercises in the voting of all proxies not otherwise assigned whereby he was enabled to change the policy and the law of the Dental Protective Association without the knowledge of the membership or an opportunity for their enforcing a protest. Neither should there be such authority to any three men as is granted to the board of directors of the Dental Protective Association whereby the board can change the law and policy of the organization on a week's notice to itself. Neither should any three men have such extraordinary power as was exercised by the board of directors of the Dental Protective Association whereby there was adopted an agreement proviously arranged between Dr. Crouse and Dr. Taggart, providing that "*No member of the Association* is to *defend or join in or contribute to the defense of any suit upon any of said patents while practicing the method under such permission from Dr. Taggart.*

By this partnership agreement the Dental Protective Association acquires 40 percent and Dr. Taggart 60 per cent. of the money collected through this agency. Many consider this alliance more dangerous to the independence and safety of the profession than was the patent situation which threatened to enslave the profession but a few years ago. To say the least, it would be unwise, unsafe, inexcusable and insanely foolish for us to rest in idle indifference while organized effort, backed by capital, seeks to tie the hands of one after another of us until the profession loses its virility, its independence, and its community of interest.

We as a profession have had the benefit of the course pursued for three years by the defense committee in the Taggart-Boynton suit, and a permanent defensive organization may utilize the information, the experience and the data accumulated and this defence committee, of which Dr. M. F. Finley, of Washington, D. C., is chairman, and its contributing supporters, will be utilized as a nucleus of value in every respect. It seems to us the part of wisdom and sane self-protection to organize, and that ordinary prudence demands and the vast interests of the profession require the most prompt canvassing and quick response possible.

The undersigned therefore invite every reader of this to join in a thorough canvas of the whole profession and enlist as members of a new permanent organization every one whose personal interest and loyalty to the profession keeps alive his sense of justice and fraternity.

In this crisis let not the stigma of injustice or of disloyalty rest upon any member of the profession.

M. F. Finley,
W. E. Boardman,
W. H. Trueman,
V. E. Turner,
E. P. Dameron,
F. W. Stiff,
F. A. Roe,
I. A. Bass,
A. J. Cottrell,
C. S. Butler,
S. H. Voyles,
H. L. Roberts,
R. Summa,
Wm. Donnally,
Wm. Carr,
R. H. Volland,
H. L. Wheeler,
E. E. Haverstick,
F. T. Breene,
W. G. Crandall,
C. W. Rodgers,
H. C. Polton,
Geo. H. Wilson.

Responses should be addressed to:—Dr. M. F. Finley,
1926 "Eye" St., Washington, D. C.

